# RE: Ultrasonic Measurement of Testicular Tumors and the Correlation with Pathologic Specimen Sizes

**DOI:** 10.1590/S1677-5538.IBJU.2015.0668

**Published:** 2016

**Authors:** Ibrahim Karademir, Zafer Demirer, Suela Karademir, Yalcın Bozkurt, Ali Guragac

**Affiliations:** 1 Department of Radiology, Eskisehir Military Hospital, Eskisehir, Turkey;; 2 Department of Urology, Eskisehir Military Hospital, Eskisehir, Turkey;; 3 Department of Radiology, Yunus Emre Hospital, Eskisehir, Turkey;; 4 Department of Radiology, Golcuk Military Hospital, Kocaeli, Turkey;; 5 Department of Urology, Tatvan Military Hospital, Bitlis, Turkey

*To the editor,*

We read with great interest the article “The value of testicular ultrasound in the prediction of the type and size of testicular tumors” by Shtricker et al. (1). They aimed to assess the correlation between ultrasound (US) findings and testicular tumor type and size. The authors concluded that the testis US findings underestimated the size in 25% of the malignant testicular lesions and 16% of the cases were proven to be benign. Thus they recommended putting into practice frozen sections for borderline cases. This study gives substantial information on this clinically relevant condition. The awareness of this diagnostic finding and its clinical results may increase the accuracy of preoperative management of the patients with testicular lesions. Thanks to the authors for this contribution.

Several medical subspecialties manage their treatments with respect to anatomic measurements. The reproducibility and accuracy of the measurements are especially crucial in radiology as important clinical decisions are often based on the assumption that radiologic measurements are accurate and any measurement differences on follow-up imagings represent a real change in size. Favorably, measurements of the tumors on images should be accurate, reproducible, and practiced in a standardized method with low rates of intra-and interobserver variability. Even so, there a lot of factors, which may affect the consistency of the measurement, including patient-dependent factors, technical factors and radiologist-dependent factors (2,3).

World Health Organization criteria (WHO) (4) and the Response Evaluation Criteria in Solid Tumors (RECIST) (5) are two widely accepted guidelines of measurement methods to obtain standardized results (6). WHO criteria recommend the measurement method on the basis of an approximation of cross-sectional area (bidimensional measurement), whereas RECIST suggests to measure only the tumor’s greatest diameter (unidimensional measurement) on a transverse plane (7).

Shtricker et al. have designed this study as a multicenter study (1). This design may increase the variabilities in the tumor measurement. However, the authors did not mention any measurement method for standardization in the study. A lot of published studies based on the variability and reproducibility of tumor measurements define their measurement methods and they generally use WHO or RECIST criteria. Therefore a measurement variability might occur due to lack of measurement standardization.

In conclusion, we strongly believe that those findings obtained from the current study will lead to further studies examining the correlation between testis US findings and histopathology. One should keep in mind that measurement standardization and comparison with pathologic specimens in optimized conditions are essential for valuable results.

*Zafer Demirer, MD* *Department of Urology,* *Eskisehir Military Hospital, Eskisehir, Turkey* *Telephone: + 90 505 462-8289* *E-mail: zaferdemirer1903@gmail.com*
